# Definite Vestibular Paroxysmia Comorbid With Definite Ménière’s Disease: A Rare Case Highlighting Diagnostic and Therapeutic Implications

**DOI:** 10.7759/cureus.92336

**Published:** 2025-09-15

**Authors:** Kento Koda, Makoto Kinoshita, Kentaro Ichijo, Naoko Ogata, Mineko Oka, Kenji Kondo, Chisato Fujimoto

**Affiliations:** 1 Otorhinolaryngology and Head and Neck Surgery, Graduate School of Medicine, University of Tokyo Hospital, Tokyo, JPN

**Keywords:** carbamazepine, endolymphatic hydrops, ménière’s disease, vertigo, vestibular paroxysmia

## Abstract

Ménière’s disease (MD) is characterized by episodic vertigo lasting 20 minutes to several hours with fluctuating sensorineural hearing loss. Vestibular paroxysmia (VP) involves brief, recurrent vertigo attacks that typically last less than one minute and are often responsive to carbamazepine. While both diseases can present with accompanying auditory symptoms during vertigo attacks, they differ in attack duration and drug responsiveness. Here, we report a case of definite MD that was subsequently complicated by definite VP.

A 46-year-old man presented with fluctuating right-sided sensorineural hearing loss and recurrent vertigo episodes lasting about one hour, consistent with definite MD. Gadolinium-enhanced MRI demonstrated significant endolymphatic hydrops in the right vestibule without neurovascular compression. Initial treatment with isosorbide improved vertigo. One year later, he developed multiple brief vertigo spells per day, each lasting about one minute and accompanied by tinnitus and nystagmus. These episodes were unresponsive to isosorbide but resolved rapidly after carbamazepine administration, leading to a diagnosis of comorbid VP.

This case illustrates the diagnostic challenge of sequential MD and VP while emphasizing the educational value of recognizing their differences in attack duration and pharmacological response in clinical practice.

## Introduction

Ménière’s disease (MD) is an inner ear disorder characterized by episodic vertigo lasting from 20 minutes to several hours, accompanied by fluctuating sensorineural hearing loss [1]. The underlying pathology is considered to be endolymphatic hydrops, which reflects abnormal accumulation of endolymph within the inner ear [2]. Treatment typically involves diuretics, lifestyle modification, or intratympanic or surgical interventions, but recurrence and progression remain frequent clinical challenges [3,4].

In contrast, vestibular paroxysmia (VP) presents with recurrent vertigo attacks lasting less than one minute, often accompanied by auditory symptoms [5]. Its pathophysiology is thought to involve neurovascular compression at the root entry zone of the vestibular nerve or hyperexcitability of demyelinated fibers [6]. A characteristic feature of VP is its favorable response to sodium channel blockers, particularly carbamazepine [5]. The diagnostic criteria for both MD and VP, as proposed in the International Classification of Vestibular Disorders consensus documents, are categorized into “definite” and “probable” types according to the fulfillment of specific clinical features [1,7].

Although both MD and VP may present with vertigo accompanied by auditory symptoms, they differ in attack duration and pharmacological responsiveness. Because of this symptomatic overlap, misdiagnosis or delayed recognition is possible, particularly in cases where both disorders coexist. VP cohorts and reviews generally exclude concomitant vestibular disorders (including Ménière’s disease), so explicit comorbidity reports are not apparent; our case - definite MD followed by definite VP - helps fill this gap [6,8].

Here, we describe a case of definite MD that was subsequently complicated by definite VP, underscoring the importance of careful evaluation of vertigo characteristics and treatment responses in distinguishing these two conditions.

## Case presentation

**Figure 1:**
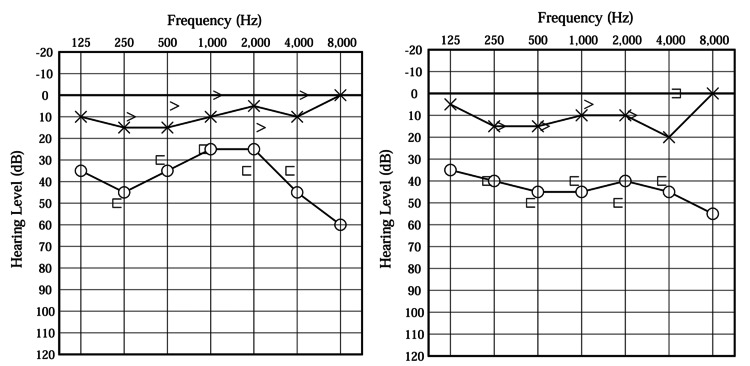
Right-sided Ménière’s disease–associated hearing fluctuation The right-sided sensorineural hearing loss progressed over time, fluctuating with each episode and eventually resulting in sustained deterioration, consistent with the fluctuating and progressive hearing loss characteristic of MD.

A 46-year-old man had experienced recurrent episodes of rotatory vertigo, each lasting approximately one hour, for about six years before his initial presentation. These vertigo attacks were consistently accompanied by fluctuating right-sided auditory symptoms, including aural fullness, tinnitus, and sensorineural hearing loss. Audiograms obtained during this period demonstrated fluctuating thresholds in the right ear, confirming the objective variability of hearing loss (Figure 1).

At the initial visit (October, Year 0), the patient presented to our clinic. Pure-tone audiometry was normal. There was no nystagmus observed on visual fixation, nor was any nystagmus induced during positional or positioning tests under infrared CCD camera examination.

One month later (November), vertigo improved after starting oral isosorbide and remained stable without medication thereafter.

**Figure 2:**
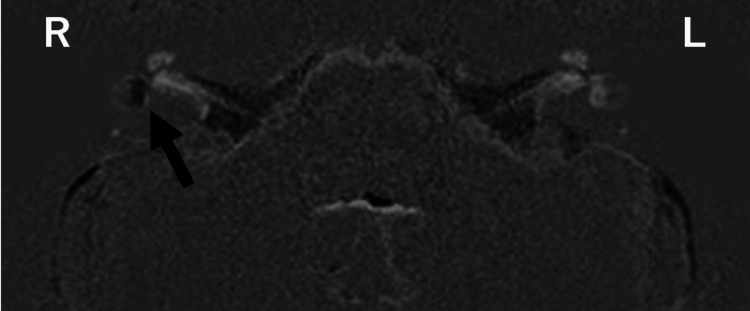
Marked endolymphatic hydrops in the right vestibule Following intravenous administration of a single dose of gadolinium-based contrast agent, inner ear MRI was performed using heavily T2-weighted cisternography, positive perilymph image (PPI), and positive endolymph image (PEI) sequences to visualize the endolymphatic space. No abnormal findings suggestive of endolymphatic hydrops were observed in either cochlea. However, the right vestibule showed marked endolymphatic hydrops occupying more than half of the entire vestibular area. No hydrops was detected in the left vestibule. No findings suggestive of neurovascular compression or nerve entrapment were observed.

Two months after the initial visit (December), MRI additionally revealed prominent vestibular hydrops in the right ear, with no evidence of neurovascular compression (Figure 2).

Three months after the initial visit (January), both the caloric test and the video head impulse test were normal. Cervical and ocular vestibular evoked myogenic potential testing (cVEMP and oVEMP testing) showed reduced responses on the right side (Table 1).

**Table 1:** Results of vestibular function tests CP: canal paresis; SPV: slow phase velocity; AR: asymmetry ratio; cVEMP: cervical vestibular evoked myogenic potential; oVEMP: ocular vestibular evoked myogenic potential; vHIT: video head impulse test Reduced VEMP responses on the right side indicate unilateral vestibular dysfunction, in line with the MD diagnosis.

Examination	Right Ear	Left Ear	Reference Value (Abnormal if…)
Caloric test (ice water, 4°C, 2 mL, 20 s)	Normal	Normal	CP >20% or SPV <10 °/s
ACS cVEMP (air-conducted sound)	Decreased response	Normal	AR >34% or absent
BCV oVEMP (bone-conducted vibration)	Decreased response	Normal	AR >27.3% or absent
vHIT (video head impulse test)	Normal	Normal	Gain <0.8 (lateral), <0.7 (vertical)

At that time, left-beating horizontal spontaneous nystagmus was observed, indicating an active phase of the disease. A diagnosis of definite right-sided MD was made based on clinical criteria. Similar episodes of nystagmus were intermittently observed during follow-up.

Approximately seven months after the initial visit (early May), he began experiencing brief episodes (approximately 1 minute) of horizontal swaying sensation several times per day (typically 5-6 times), which worsened with upward gaze. At the same time, newly observed nystagmus and worsening tinnitus were noted on examination. The episodes tended to cluster at similar times each day, and during attacks, the patient described brief episodes of visual instability.

The patient resumed isosorbide on his own, but the symptoms persisted. Two weeks later, he returned to our clinic. Suspecting comorbid VP, we initiated oral carbamazepine therapy, which promptly resolved his symptoms. He has since been followed for four months without recurrence of vertigo attacks.

## Discussion

In the present case, the patient had already been diagnosed with definite MD, based on repeated episodes of vertigo accompanied by fluctuating sensorineural hearing loss in the right ear. Approximately one year after the initial visit, he developed brief, recurrent episodes of vertigo lasting approximately one minute and often occurring multiple times per day. These episodes were distinct from the typical MD attacks. Notably, these new symptoms were not accompanied by changes in hearing and responded dramatically to low-dose carbamazepine. Based on these findings, a diagnosis of coexisting definite VP was made.

Short, recurrent attacks of vertigo brought on by VP are thought to be due to neurovascular compression at the root entry of the vestibular nerve and hyperexcitability of the vestibular nerve [5,6]. These attacks respond well to sodium channel blockers, such as carbamazepine and oxcarbazepine [7]. Although no obvious neurovascular compression was detected on MRI, mechanisms such as functional compression in this case, demyelination, or axonal hyperexcitability have been reported to underlie the pathophysiology of VP even in the absence of radiological findings [8].

Both MD and VP are relatively common diseases that primarily affect middle-aged and older individuals. However, VP occurring after MD is rare. It is difficult to determine whether the two diseases occur together by coincidence or if they share overlapping physiological mechanisms. Previous VP cohorts and reviews have excluded patients with concomitant vestibular disorders such as MD. Therefore, explicit reports of MD-VP comorbidity are lacking. Our case highlights this rare coexistence and provides clinical evidence to fill this gap [6,8].

Patients with MD are considered to experience recurrent episodes of vertigo caused by endolymphatic hydrops. Elevated levels of proinflammatory cytokines have been reported in the perilymphatic fluid and systemic circulation of MD patients, suggesting a chronic inflammatory state [9,10]. Additionally, functional imaging studies have demonstrated central hyperresponsiveness in MD, revealing abnormal activation patterns in vestibular processing regions [11]. On the other hand, VP is thought to result primarily from hyperexcitability of the peripheral vestibular nerve, most commonly due to neurovascular compression or, less frequently, demyelination [5].

Based on these findings, repeated episodes of endolymphatic hydrops in MD may chronically stimulate the cochlear and vestibular nerve terminals. This stimulation may increase the excitability of sensory neurons and cause fluctuations in activation threshold in the central vestibular pathways. This hyperexcitability may lead to exaggerated responses to minor vestibular stimuli and give rise to the brief paroxysmal vertigo characteristic of VP. Further studies are needed to clarify the mechanisms underlying the coexistence of these two diseases.

## Conclusions

In a patient with definite MD, the subsequent emergence of brief recurrent vertigo responsive to carbamazepine indicated comorbid VP. Recognizing such coexistence is essential to avoid misdiagnosis or treatment delay, although this report is limited by being a single case and by the absence of radiological evidence for neurovascular compression.
